# Is there an association between COVID-19 mortality and Human development index? The case study of Nigeria and some selected countries

**DOI:** 10.1186/s13104-022-06070-8

**Published:** 2022-05-21

**Authors:** Sanyaolu Alani Ameye, Temitope Olumuyiwa Ojo, Tajudin Adesegun Adetunji, Michael Olusesan Awoleye

**Affiliations:** 1 Department of Otorhinolaryngology, Obafemi Awolowo University, Ile-Ife, Osun Nigeria; 2 Department of Community Health, Obafemi Awolowo University, Ile-Ife, Osun Nigeria; 3 Department of Internal Medicine, Obafemi Awolowo University Teaching Hospitals Complex, Ile-Ife, Osun Nigeria; 4 African Institute for Science Policy and Innovation, Obafemi Awolowo University, Ile-Ife, Nigeria

**Keywords:** COVID-19, Nigeria, Case Fatality Rate, Metrics, Human Development Index

## Abstract

**Objectives:**

We assessed Case Fatality Rate (CFR) of COVID-19 as an indicator to situate the performance of Nigeria relative to other selected countries. We obtained case fatality rates of different countries from data sets available from open-sources. The CFRs were calculated as the rate of deaths compared with total cases. The values were compared with Nigeria’s COVID-19 CFR. Other relevant statistical comparisons were also conducted.

**Results:**

The worst performing countries with regards to CFR in descending order were Yemen (19.5%), Peru (9.0%) Mexico (7.6%), Sudan (7.4%) and Ecuador (6.3%) while the best performing nations were Bhutan (0.11%), Burundi (0.19%), Iceland (0.20%), Laos (0.21%) and Qatar (0.25%). The CFR of Nigeria was 1.39% which falls below the 50th percentile. Other comparison done showed significant difference in the CFR values between countries similar to Nigeria and countries that are dissimilar when HDI is used. (Mann–Whitney U test 126.0, p = 0.01). The trend of the CFR in Nigeria showed a steady decline and flattening of the CFR curve which does not seem to be affected by the spikes in the daily declared cases.

**Supplementary Information:**

The online version contains supplementary material available at 10.1186/s13104-022-06070-8.

## Introduction

Since the declaration of the novel coronavirus disease (COVID-19) as a global pandemic on March 11, 2020 [1], it became a clear and present danger to developing countries like Nigeria. In the early days of the pandemic the health and economic effects of COVID-19 were obvious in the high income countries of Europe and America and it was expected that the effect of the disease may be more marked in low income countries [2]. At the onset, many public safety and non-pharmacologic measures were instituted to limit the spread of the virus. These measures included the closure of international borders, lockdown order within countries, stay-at-home order for citizens, restriction of public gatherings as well as the ban on non-essential travel [3–5]. These aforementioned measures have since been relaxed in many countries of the world. However, the non-pharmacological preventive measures which are still in force to varying degrees include the use of facemasks, frequent hand washing with soap and running water, sanitizing with alcohol-based hand rub, and physical/social distancing [6, 7].

Most countries of the world are affected by COVID-19 with almost 500 million cases and over 6 million deaths recorded [9]. SARS-CoV2, the implicated virus in COVID-19, is highly infectious with a median basic reproduction rate (R_0_) of up to 5.7 [8]. This makes the community transmission of COVID-19 rapid especially in places where control measures are poorly implemented. Although fewer cases and deaths were reported from low-middle income nations, the economic constraints occasioned by the pandemic are apparent [10]. For instance, in Nigeria, throughout the four waves of the COVID-19 epidemic, a total of 255,516 cases as of April 4, 2022 were reported [11]. However, arising from the pandemic, the country experienced the largest economic contraction in a decade [12].

Just like some other low-income/lower middle income countries, Nigeria's healthcare system is plagued with poor infrastructure [13] and personnel flight [14] which may have been worsened by the pandemic. Hence, it is pertinent to examine the disease trend and country performance in the world’s most populous black nation after about two years of the outbreak [15], considering the postulations that the disease may behave differently across ethnicities with blacks being worse-hit than the other races [16–18].

However, for a novel disease such as this, there is the need for a metric that can be used to assess trends/performance using country-level data among nations with similar development indices. One of such widely used comprehensive metric is the excess mortality, and the derivative P-score which provide information on the number of deaths from all causes during a crisis above and beyond what we would have expected to see under ‘normal’ conditions [19]. However, not all countries have the infrastructure and capacity to register and report all deaths.

The United Nation (UN) estimates that, in “normal” times, only two-thirds of countries register at least 90% of all deaths that occur, and some countries register less than 50% and as low as under 10% deaths [20]. During the pandemic we expect this reporting to be lower. Even in settings where every mortality gets reported ordinarily, some delays were occasioned by the pandemic [21] In Low/Middle Income Countries (LMIC) like Nigeria, undercounting of mortality is an issue and thus the country has no information that is required to derive the excess mortality and P-Score. The use of the mortality rate cannot be of much value in comparing the country with others because it may not represent the true picture considering the fact that this metric is affected by the population size and the significantly varied rate of testing among countries [22].

We therefore present the simple Case Fatality Rate (CFR), which is a measurement that represents the proportion of deaths within a defined population of interest, i.e. the percentage of cases that result in death. [23, 24]. We believe this metric is a work-around that can be used to track the performance in a setting like ours. This study compared the performance of the country against different groups of nations using the case fatality rate (CFR). This may provide useful information to situate the burden of COVID-19 in Nigeria.

## Main text

### Methods

This is a secondary data analysis that compared disease severity between Nigeria and some other countries. The dataset was obtained from the “Our World In Data” (OWID) COVID-19 database [25] and the Human Development Index (HDI) database [26]. For the COVID-19 dataset, the cut-off date was the 26th of November, 2021. The required data was acquired, parsed, cleansed, and aggregated using the Python Data Science Environment [27] with essential packages such as Pandas^®^ Library [28].

The point case fatality rates (%) were generated from the OWID dataset as a derivative of the total death and total cases. These case fatality estimates were derived from the currently available data as of 26th November 2021. Countries with total cases less than 1000 were however filtered out from this analysis.

From the modified OWID dataset, the trend of new cases and case fatality rates were also calculated during the period for the country.

### Performance of Nigeria in relation with similar and dis-similar countries on HDI

The case fatality rate of Nigeria was examined with neighbouring countries (Niger, Chad, Cameroon, Togo, Ghana, Benin) and continental averages.

To obtain the countries that are similar and dissimilar (countries at a maximal distance) to Nigeria in HDI, Ward’s minimum-variance hierarchical clustering analysis was conducted [29] using an agglomerative (bottom-up Hierarchal Clustering up) approach and Ward’s linkage on the HDI dataset. The metric of interest, i.e., the CFR was then compared between these countries and other groups of countries. The results are summarized using frequencies as well as proportions and are presented as charts.

## Results

**Fig. 1:**
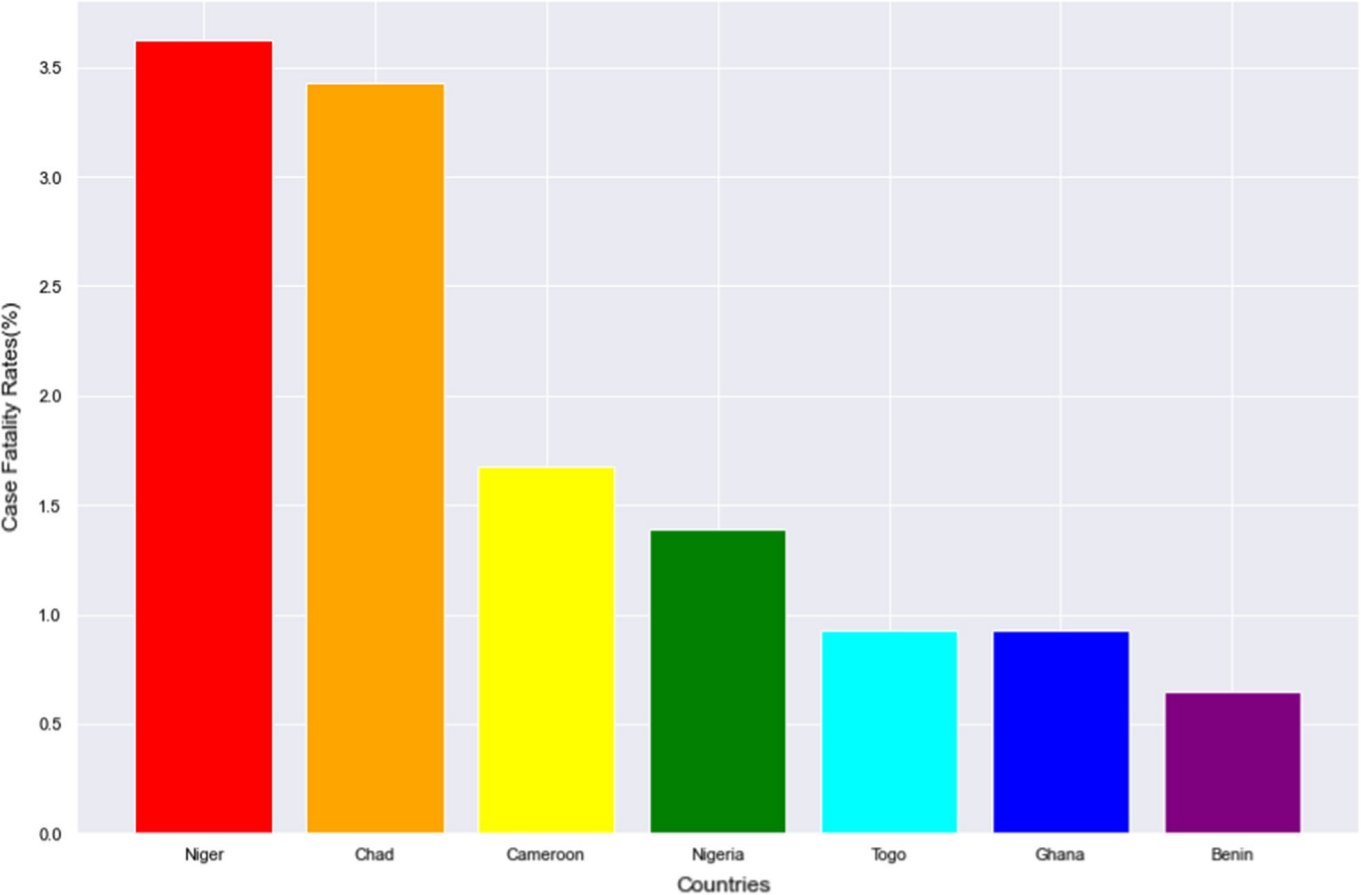
The Case Fatality Rates of Nigeria and Neighbouring Countries

**Fig. 2:**
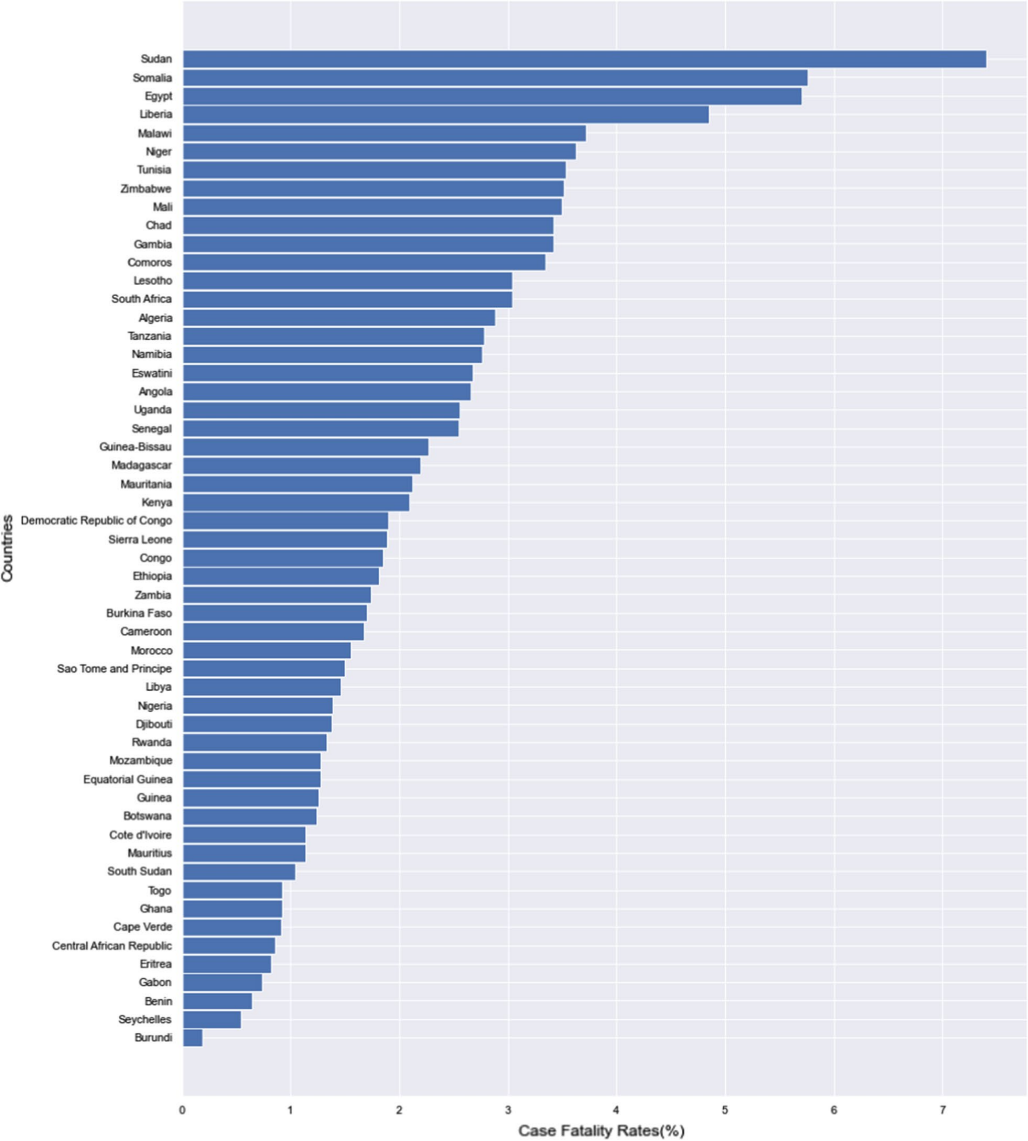
Case Fatality rates in African countries

**Fig. 3:**
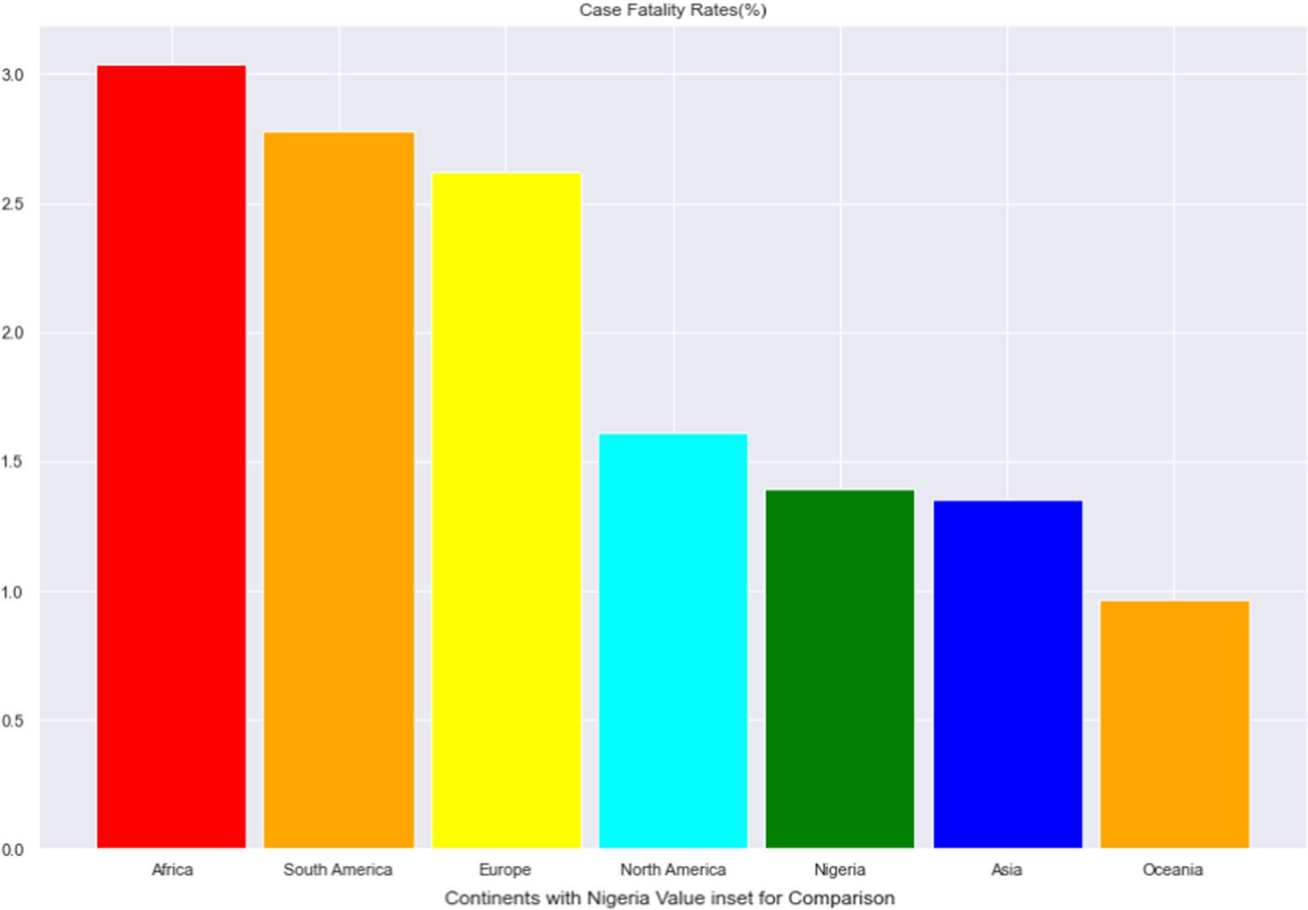
The Average Case Fatality Rates across continents with the Nigerian value inset for comparison

As of November 26th, 2021 the worst 5 nations with regards to CFR were Yemen (19.5%), Peru (9.0%) Mexico (7.6%), Sudan (7.4%) and Ecuador (6.3%) while the best nations with low CFR were Bhutan (0.11%), Burundi (0.19%), Iceland (0.20%), Laos (0.21%) and Qatar (0.25%). Nigeria at this time has had 213,883 cases with 2,063 deaths giving a CFR of 1.39%. Figures 1, 2 and 3 show these rates in comparison with neighbouring countries, African Countries and Continental Averages respectively.

Based on the HDI using hierarchical clustering, six countries (Angola, Sierra Leone, Central African Republic, Ivory Coast, and Chad) were found to be similar to Nigeria. Additional file 1: Fig. S1 shows the CFR of these countries including Nigeria.

Twenty-two countries (Australia, Austria, Belgium, Canada, Cuba, Denmark, Finland, France, Germany, Iceland, Israel, Japan, South Korea, Luxembourg, Malta, Netherlands, New Zealand, Norway, Sweden, Switzerland, United Kingdom, United States) were found to be at a maximum distance with regards to similarity which implies that they were quite dissimilar to Nigeria on HDI. Additional file 1: Figure S2 shows the general comparison of CFR among countries with similar HDIs to Nigeria and countries with dissimilar HDIs. The Mann–Whitney U test (W = 126.0, p = 0.01).

Additional file 1: Fig. S3 shows the daily declared new cases and the trend of the Case Fatality Rates over the period under review.

## Discussions

The OWID database is a useful and rapidly evolving database that provides great insight into the data related to COVID-19 generally and as it affects Nigeria, the most populous black nation [30]. The choice of CFR as a metric for measuring the performance of this disease and perhaps the health system was justified earlier.

Our main focus however, is on how Nigeria fared in comparison to other countries and regions. Nigeria at the time of our analysis has a CFR value that falls in the midrange of the CFR values of different countries of the World. When compared with the countries six cultural/geographical neighbours, the country ranked 4th with Togo, Ghana and Benin having lower CFR. Comparing the CFR in Nigeria to continental averages showed that while Africa still has the worst continental value, the CFR in Nigeria is only higher than that of Asia and Oceania. It is pertinent to note that the SARS CoV2 virus originated from Asia [31] and more insight will be obtained if we are to drill down on the trend of the CFR on this continent.

When these countries with similar HDI were compared with Nigeria, the country ranked 5 out of 7 with Cote d’Ivoire and Central African Republic having a lower CFR. When these two groups of countries (low and high HDI) were compared our finding showed that the CFR is expectedly significantly higher in countries with low HDI. This perhaps suggests that factors such as health facility infrastructure and capacity may have a larger influence on the CFR.

Looking at the trend, it became apparent that Nigeria had spikes in daily new cases mainly in July 2020, January to February 2021 and more recently October 2021 (rather transient spike). With regards to the CFR, the trend chart gives insight into the fact there has been a steady decline in the CFR with the period of spikes not affecting the trend of this metric. The trend information gives more insight than point calculations of CFR given that there is expectedly a lag between becoming infected and outcome.

The implication of the decreasing CFR may suggest growing herd immunity to the SARS CoV2 virus in the general populace especially in the context of widespread community transmissions. However, this work and available data lack the necessary information to test this theory.

From our findings, Yemen presents a dismal performance with regards to the CFR and this has been grossly underreported in the media and academic literature. Perhaps, the sectarian war with its attendant humanitarian crises could be responsible for this [32]. In this same group of the top five countries with the worst CFR, Peru was a distant second to Yemen followed by Mexico, Sudan and Ecuador. While the scenario that applies to Yemen may apply to Sudan there appears to be no obvious reason why Mexico, Peru and Ecuador feature in this group. However, it is interesting to note that they are all South American Countries and further inquiry may yield interesting findings.

Similarly, the best performing countries however also feature some countries that elicit surprises. While it is not surprising that Iceland made this list considering her very high HDI, finding countries like Burundi, Bhutan, and Laos in this group is perhaps counterintuitive. For example, Burundi, a landlocked country in East Africa, has had her fair share of conflicts with well-documented attendant humanitarian and health-related problems [33]. It is therefore not expected to be among countries with the lowest CFR. Similarly, countries like Bhutan (also a landlocked country) and Laos can be regarded as developing countries [34, 35]. We explored the link between being landlocked and the possibility of limiting exposure to visitors and the different variants of the SARs CoV2 virus but Bhutan enjoys robust interactions with foreigners due to the recreational mountaineering activities [36], though these recreational activities may have been slowed down by the country response which included two lock-downs implemented in the country so far [37].

## Conclusion

It is important to be cognizant of the fact that the COVID-19 pandemic is still ongoing and our understanding will continue to deepen as we acquire more data. Although it appears that Nigeria has lower case fatality rates compared to other countries with similar HDI, we make no claim to an immutable conclusion from our effort in looking at the relevant data. Future studies should focus on the factors that may be responsible for the apparently low case fatality rates in Nigeria.

## Limitations

At the beginning of the pandemic, most (African) countries had limited testing capacities [25] and this may have affected the number of cases and death reported. Similarly, there may be cases of under-reporting by some countries [38] and this may also affect the official case burden recorded for the affected countries. We can only hope that as more data is gathered on this pandemic and performances as highlighted here are deeply studied, more actionable knowledge will be derived.

## Supplementary Information

**Additional file 1: Figure S1.** The ranking of the country with regards to the CFR among countries with similar HDI to Nigeria. **Figure S2.** Comparison of CFR among countries with similar HDIs to Nigeria and countries with dissimilar HDIs. **Figure S3.** The Trend of the Daily New Cases and Case Fatality Rate in Nigeria

## Data Availability

The dataset used for this study is available at https://ourworldindata.org/coronavirus-source-data

## Abbreviations

CFR: Case Fatality Rate
COVID-19: Corona Virus Disease 2019
HDI: Human Development Index
LMIC: Low/Middle Income Countries
OWID: Our World in Data
SARS-CoV2: Severe Acute Respiratory Syndrome- Coronavirus 2
UN: United Nations
