# Supplementary material for: Is there an association between COVID-19 mortality and Human development index? The case study of Nigeria and some selected countries
Source: BMC Res Notes. 2022 May 21;15:186. doi: 10.1186/s13104-022-06070-8 (PMC9123789; doi:10.1186/s13104-022-06070-8)
Supplement: Supplementary file 1 — Additional file 1: Figure S1. The ranking of the country with regards to the CFR among countries with similar HDI to Nigeria. Figure S2. Comparison of CFR among countries with similar HDIs to Nigeria and countries with dissimilar HDIs. Figure S3. The Trend of the Daily New Cases and Case Fatality Rate in Nigeria [file 13104_2022_6070_MOESM1_ESM.docx]

Additional file 1: figures


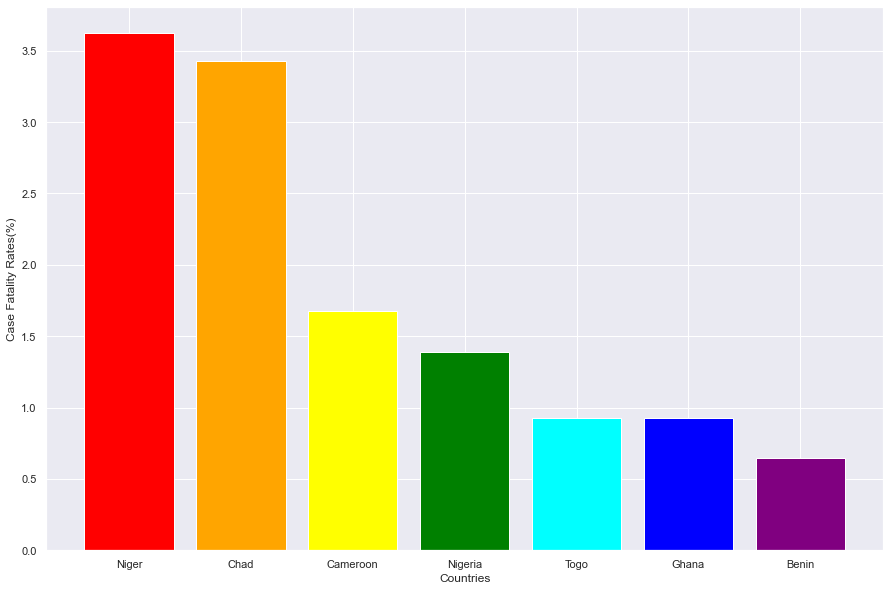


Figure S1. The ranking of the country with regards to the CFR among countries with similar HDI to Nigeria.


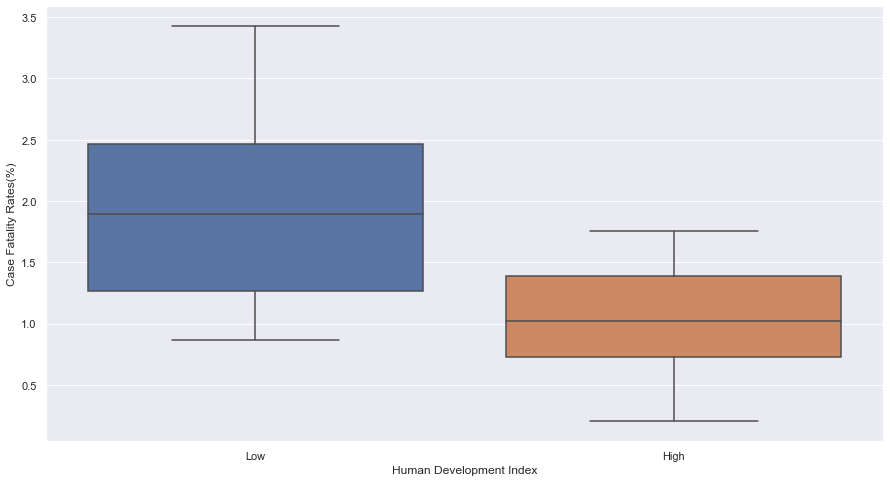


Figure S2. Comparison of CFR among countries with similar HDIs to Nigeria and countries with dissimilar HDIs.


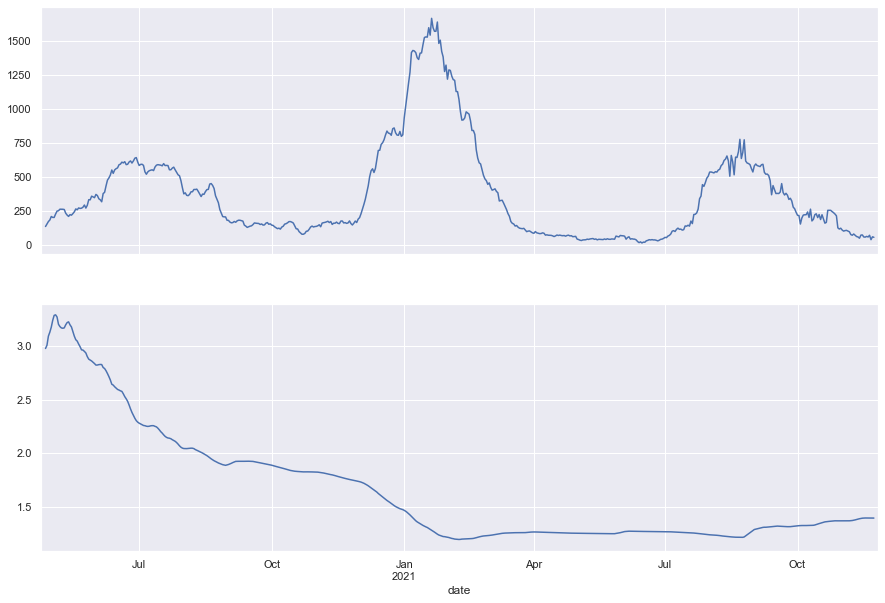


Figure S3. The Trend of the Daily New Cases and Case Fatality Rate in Nigeria
